# Thymic Stromal-Cell Abnormalities and Dysregulated T-Cell Development in IL-2-Deficient Mice

**DOI:** 10.1155/1998/19567

**Published:** 1998

**Authors:** Tannishtha Reya, Hamid Bassiri, Renée Biancaniello, Simon R. Carding

**Affiliations:** 1 Department of Microbiology University of Pennsylvania School of Medicine 303A Johnson Pavilion Philadelphia PA 19104 USA; 2 Department of Microbiology and Immunology Howard Hughes Medical Institute University of California 513 Parnasus Avenue San Francisco CA 94143 USA

**Keywords:** Cytokines, thymus, T-cell development, stromal cells

## Abstract

The role that interleukin-2 (IL-2) plays in T-cell development is not known. To address this issue, we have investigated the nature of the abnormal thymic development and autoimmune disorders that occurs in IL-2-deficient (IL-2^–/–^) mice. After 4 to 5 weeks of birth, IL-2^–/–^ mice progressively develop a thymic disorder resulting in the disruption of thymocyte maturation. This disorder is characterized by a dramatic reduction in cellularity, the selective loss of immature CD4^-^8^-^ (double negative; DN) and CD4^+^8^+^ (double positive; DP) thymocytes and defects in the thymic stromal-cell compartment. Immunohistochemical staining of sections of thymuses from specific pathogen-free and germ-free IL-2^–/–^ mice of various ages showed a progressive ,loss of cortical epithelial cells, MHC class II-expressing cells, monocytes, and macrophages. Reduced numbers of macrophages were apparent as early as week after birth. Since IL-2^–/–^ thymocyte progenitor populations could mature normally on transfer into a normal thymus, the thymic defect in IL-2^–/–^ mice appears to be due to abnormalities among thymic stromal cells. These results underscore the role of IL-2 in maintaining functional microenvironments that are necessary to support thymocyte growth, development, and selection.


*Developmental Immunology*, 1998, Vol. 5, pp. 287–302
Reprints available directly from the publisher
Photocopying permitted by license only

© 1998 OPA (Overseas Publishers Association)
N.V. Published by license under the
Harwood Academic Publishers imprint,
part of the Gordon and Breach Publishing Group
Printed in Malaysia

### Thymic Stromal-Cell Abnormalities and Dysregulated T-Cell Development in IL-2-Deficient Mice

TANNISHTHA REYA[†] HAMID BASSIRI, RENÉE BIANCANIELLO and SIMON R. CARDING[*]

[a]*Department of Microbiology, University of Pennsylvania School of Medicine, 303A Johnson Pavilion, Philadelphia, PA 19104*

*(Received 12 June 1997; In final form 22 August 1997)*

The role that interleukin-2 (IL-2) plays in T-cell development is not known. To address this issue, we have investigated the nature of the abnormal thymic development and autoimmune disorders that occurs in IL-2-deficient (IL-$2^{-/-}$) mice. After 4 to 5 weeks of birth, IL-$2^{-/-}$ mice progressively develop a thymic disorder resulting in the disruption of thymocyte maturation. This disorder is characterized by a dramatic reduction in cellularity, the selective loss of immature $CD4^{-}8^{-}$ (double negative; DN) and $CD4^{+}8^{+}$ (double positive; DP) thymocytes and defects in the thymic stromal-cell compartment. Immunohistochemical staining of sections of thymuses from specific pathogen-free and germ-free IL-$2^{-/-}$ mice of various ages showed a progressive ,loss of cortical epithelial cells, MHC class II-expressing cells, monocytes, and macrophages. Reduced numbers of macrophages were apparent as early as 1 week after birth. Since IL-$2^{-/-}$ thymocyte progenitor populations could mature normally on transfer into a normal thymus, the thymic defect in IL-$2^{-/-}$ mice appears to be due to abnormalities among thymic stromal cells. These results underscore the role of IL-2 in maintaining functional micro-environments that are necessary to support thymocyte growth, development, and selection.

*Keywords:* Cytokines, thymus, T-cell development, stromal cells

### INTRODUCTION

Thymocyte development is dependent on the intimate association with stromal cells and the cytokine milieu of the thymus (reviewed in Carding and Reya, 1994). Among the cytokines that are normally produced within the thymus during T-cell development, IL-2 has been the most intensively studied. The expression of functional IL-2Rs by developing thymocytes (Skinner, et al., 1987; Jenkinson et al., 1987; Waanders et al., 1989, 1990; Zuniga-Pflucker et al., 1990), and the ability of anti-IL-2R antibodies to disrupt thymocyte growth and/or proliferation *in vivo* (Tentori et al., 1988; Zuniga-Pflucker and Kruisbeek, 1990; Tanaka et al., 1992) provide strong evidence for a direct role for IL-2 in thymocyte development.

[*]Corresponding author. Tel.: 215-573-3022, Fax: 215-573-9068, E-mail: carding@mail.med.upenn.edu.
[†]Present address: Department of Microbiology and Immunology Howard Hughes Medical Institute, University of California, 513 Parnasus Avenue, San Francisco, CA 94143.

Recent studies analyzing thymocyte development in IL-2-deficient (IL-$2^{-/-}$) mice have provided more direct evidence for a requirement for IL-2 for normal thymocyte development.

Although the initial analysis of thymocyte development in neonatal IL-$2^{-/-}$ mice did not detect any changes in the cellularity or composition of thymocytes (Schorle et al., 1991), these animals subsequently develop a fatal autoimmune disorder characterized by hyperactivation of peripheral T cells, lymphadenopathy, splenomegaly, hemolytic anemia, and colitis (Sadlack et al., 1993; Horak et al., 1995). Subsequent studies of IL-$2^{-/-}$ mice bred onto the athymic *nu/nu* background and RAG-$2^{-/-}$ mice reconstituted with IL-$2^{-/-}$ bone marrow have shown that the immunopathology in these mice depends on the intrathymic differentiation of T cells carrying the IL-2 mutation (Krämer et al., 1995). Other studies identifying phenotypic changes in subsets of thymocytes (Hanke et al., 1994; Lúdvíksson et al., 1997), or abnormal cytokine production (Lúdvíksson et al., 1997) by IL-$2^{-/-}$ thymocytes also implicate defects in thymic development as the underlying basis of disease pathology in these mice. Finally, our recent finding that IL-$2^{-/-}$ mice kept in a germ-free environment also develop lymphoid hyperplasia and autoimmunity (Contractor et al., in press) is also consistent with a defect in central and/or peripheral tolerance being responsible for disease rather than an uncontrolled peripheral T-cell response to environmental antigens. Although the evidence in favor of IL-2 being involved and required for thymocyte growth, survival, and/or development is compelling; the questions of how IL-2 influences these processes and what is the underlying basis of abnormal thymocyte development in IL-$2^{-/-}$ mice remain unanswered.

Here, we have investigated the nature of defective thymocyte development in IL-$2^{-/-}$ mice. The possibility that IL-2 may regulate T-cell development indirectly by influencing stromal components of the thymic microenvironment has been ignored or overlooked in many of the previous studies. To address the role of IL-2 in thymic development, we have determined what the consequences of IL-2 deficiency are on the growth and development of thymocytes and thymic stromal cells. The results of our analysis show that the disruption of T-cell development in IL-$2^{-/-}$ mice is related to changes in thymic architecture and deficiencies in specific stromal-cell populations.

### RESULTS

Although the autoimmune disorder that develops in IL-$2^{-/-}$ mice appears to arise as a consequence of dysregulated T-cell development (Krämer et al., 1995; Lúdvíksson et al., 1997), thymic development in these animals has, to date, not been studied in detail. Our approach, therefore, has been to characterize and identify deficiencies and/or defects within the lymphoid and nonlymphoid populations present within the thymi of IL-$2^{-/-}$ mice.

### IL-$2^{-/-}$ Thymocytes Have an Unusual Phenotype

Where as 2- to 4-week-old IL-$2^{-/-}$ mice had normal numbers and distribution of thymocytes as reported previously (Schorle et al., 1991), the thymuses of older mice progressively decreased in size and by 6 to 8 weeks of age contained up to twentyfold fewer cells than those of IL-$2^{+/+}$ or IL-$2^{+/-}$ littermates (Table I). Since the results of the analysis of IL-$2^{+/+}$ and IL-$2^{+/-}$ thymocytes were equivalent, only those obtained from IL-$2^{+/+}$ mice are shown throughout this study. The loss of cells was primarily attributable to a reduction in the number of immature thymocytes, particularly DP cells (Table I and Fig. 1). A significant, albeit less dramatic, reduction (approximately two fold) in the number of CD4 and CD8 SP thymocytes was also apparent. Consistent with the loss of the most immature thymocytes was the reduced number of $CD3^-$, DN thymocytes in the thymuses of IL-$2^{-/-}$ mice (Table I). The majority of DN thymocytes present expressed high levels of CD3 ($CD3^{hi}$) consistent with there being more mature thymocytes (Table I and Fig. 2). This proportional increase in the number of $CD3^+$ cells was shown not to be due to the expansion of $\gamma\delta$ T cells since the frequency of $\gamma\delta$ cells in the thymuses of IL-$2^{-/-}$ mice of all ages (3 to

TABLE I Thymus Cellularity and Composition in IL-2$^{-/-}$ Mice

| IL-2 genotype | Age (*w*) | *n* | Cell number (× $10^6$) Total | DN, $CD3^-$ | DN, $CD3^+$ | % thymocytes[a] DN | DP | CD4-SP | CD8-SP |
|---|---|---|---|---|---|---|---|---|---|
| +/+ | 4 | 5 | 91 ± 3 | 2.8 ± 1.0 | 1.2 ± 0.4 | 3 ± 1 | 84 ± 15 | 9 ± 3 | 5 ± 2 |
| −/− | 4 | 6 | 123 ± 15 | 2.7 ± 0.5 | 0.9 ± 0.5 | 3 ± 2 | 78 ± 10 | 10 ± 4 | 8 ± 3 |
| +/+ | 6 | 7 | 106 ± 12 | 2.2 ± 0.2 | 1.0 ± 0.3 | 3 ± 2 | 83 ± 6 | 8 ± 2 | 6 ± 3 |
| −/− | 6 | 8 | 79 ± 13 | 1.9 ± 0.3 | 1.3 ± 0.6 | 4 ± 2 | 64 ± 18 | 19 ± 6 | 12 ± 4 |
| +/+ | 8 | 5 | 113 ± 15 | 1.7 ± 0.5 | 0.7 ± 0.2 | 2 ± 2 | 84 ± 9 | 8 ± 3 | 6 ± 3 |
| −/− | 8 | 6 | 6 ± 3 | 0.01 ± 0.01 | 0.1 ± 0.1 | 2 ± 2 | 3 ± 5 | 51 ± 17 | 44 ± 12 |
| +/+ | 10 | 8 | 104 ± 19 | 2.3 ± 1.2 | 0.8 ± 0.2 | 3 ± 2 | 80 ± 8 | 9 ± 3 | 6 ± 3 |
| −/− | 10 | 4 | 4 ± 3 | 0.01 ± 0.01 | 0.1 ± 0.1 | 3 ± 2 | 5 ± 3 | 50 ± 10 | 42 ± 11 |
| *Germ-free mice* | | | | | | | | | |
| +/+ | 6 | 5 | 71 ± 14 | 2.4 ± 0.2 | 0.8 ± 0.4 | 4 ± 2 | 86 ± 12 | 6 ± 3 | 4 ± 2 |
| −/− | 6 | 6 | 79 ± 20 | 3.4 ± 0.3 | 0.5 ± 0.2 | 5 ± 3 | 81 ± 9 | 9 ± 5 | 5 ± 3 |
| +/+ | 8 | 3 | 84 ± 7 | 3.4 ± 0.2 | 0.8 ± 0.3 | 5 ± 2 | 81 ± 8 | 9 ± 3 | 5 ± 2 |
| −/− | 8 | 5 | 72 ± 9 | 1.8 ± 0.4 | 0.4 ± 0.2 | 3 ± 2 | 86 ± 10 | 7 ± 4 | 4 ± 3 |
| +/+ | 10 | 4 | 88 ± 11 | 2.1 ± 0.1 | 0.5 ± 0.3 | 3 ± 1 | 85 ± 4 | 8 ± 3 | 4 ± 2 |
| −/− | 10 | 4 | 31 ± 10 | 0.7 ± 0.2 | 0.6 ± 0.2 | 4 ± 2 | 54 ± 12 | 27 ± 5 | 15 ± 4 |

Determined by three-color flow cytometry.

8 weeks) was comparable to that seen in wild-type mice (Fig. 2). In contrast to DN cells from IL-2$^{+/+}$ thymuses, a higher proportion of IL-2$^{-/-}$ DN cells expressed CD44 (Fig. 2). Since most of these cells also express high levels of CD3, CD44 expression is unlikely in this case to be a marker of immaturity (Godfrey and Zlotnik, 1993) and reflects the accumulation of progenitor cells unable to develop further in the absence of IL-2. Although we have not carried out a more detailed phenotypic analysis of IL-2$^{-/-}$ DN thymocyte subsets, the fact that the number of $CD3^-CD4^-CD8^-$ progenitor cells in IL-2$^{-/-}$ mice was never greater than that seen in wild-type littermates (Table I) is consistent with there being no block in the development of IL-2$^{-/-}$ T-cell progenitors. Instead, expession of CD44 may reflect aberrant activation of $CD3^+$ DN cells in the IL-2$^{-/-}$ thymuses. This interpretation is supported by the finding that a higher proportion of mature splenic and lymph-node T cells in IL-2$^{-/-}$ mice express these activation-associated antigens when compared to the same T-cell populations in wild-type mice (data not shown; Sadlack et al., 1995). Moreover, in both the spleen and the lymph node, there was a three- and fourfold expansion of both the CD8 and CD4 T cells compared to the T cells in wild-type mice similar to that described by Sadlack et al., (1995).

### IL-2$^{-/-}$ Thymocyte Defect Is Rescued After Transfer to a Normal Thymic Microenvironment

To determine whether the loss of DN and DP thymocytes in IL-2$^{-/-}$ mice were due to an inherent requirement for IL-2 and an intrinsic developmental defect, or disruption in the microenvironment, 1 to 2 × $10^5$ TN thymocytes from 4-week-old wild-type and IL-2$^{-/-}$ mice (Thy $1.2^+$) were transferred to the thymus of wild-type mice. At this age, the IL-2$^{-/-}$ thymus is intact with cellularity and composition of thymocytes similar to that of IL-2$^{+/+}$ littermates (Table 1). The selected thymocytes contained less than 2% $CD4^+$, $CD8^+$, or Ig-coated cells and between 1% and 2% expressed low levels of CD3. The estimated cell recovery was approximately 85%. One to two hundred thousand cells were injected into each thymic lobe of sublethally irradiated Thy $1.1^+$ C57BL.6 mice. At 4 to 5 weeks after transfer, none of the animals ($n$ = 4) receiving IL-2$^{-/-}$ thymocytes showed any clinical signs of disease, and at necropsy, the thymus, spleen, lymph nodes, and colon were

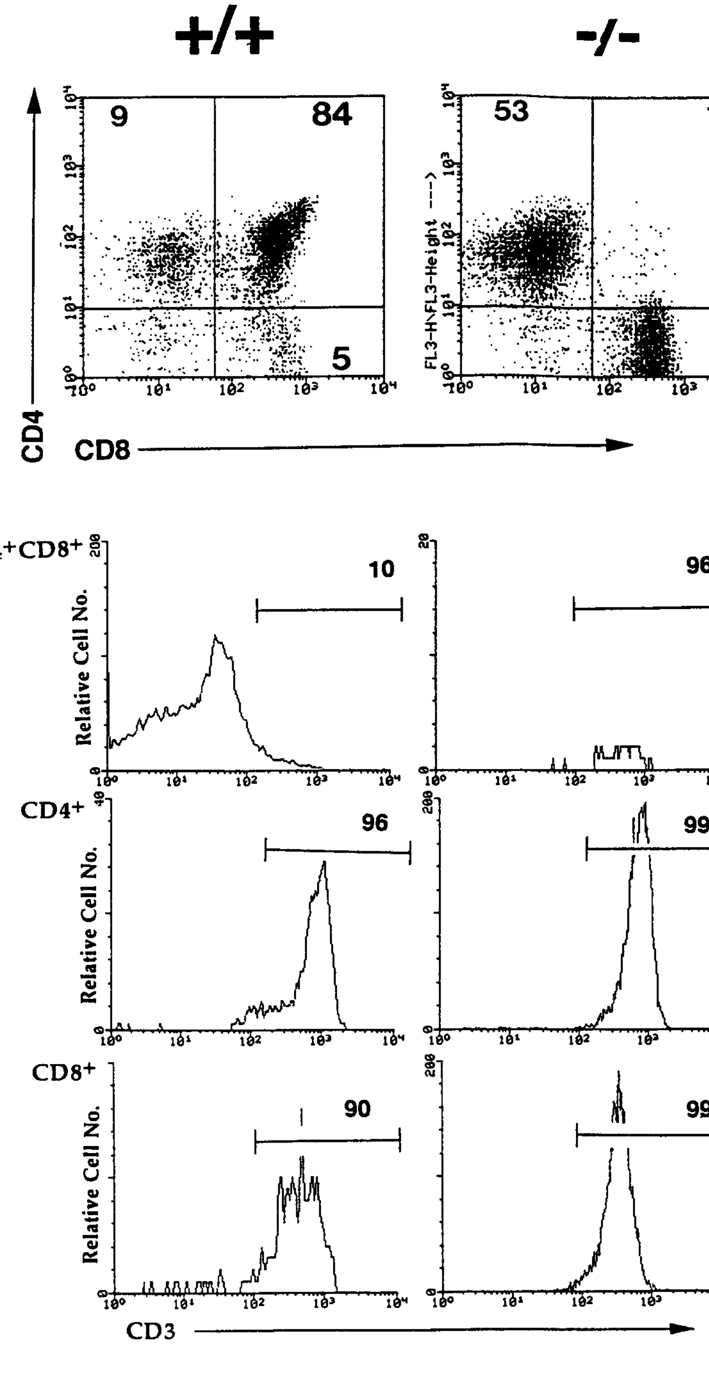

FIGURE 1 Loss of immature thymocytes in IL-$2^{-/-}$ mice. Thymocytes from 5-week-old SPF IL-$2^{-/-}$ (−/−) or IL-$2^{+/+}$ (+/+) littermates were stained with PE-conjugated anti-CD4, FITC-conjugated anti-CD8 (upper dot plots), and biotinylated anti-CD3 and strepavidin-Red 670. The values shown represent the frequency of positive cells in each quadrant. The lower histograms show the level of CD3 expression by different subsets of CD4- and CD8-expressing thymocytes. The values shown represent the frequency of positive cells. The results shown are representative of those obtained from eight IL-$2^{-/-}$ and seven IL-$2^{+/+}$ mice.

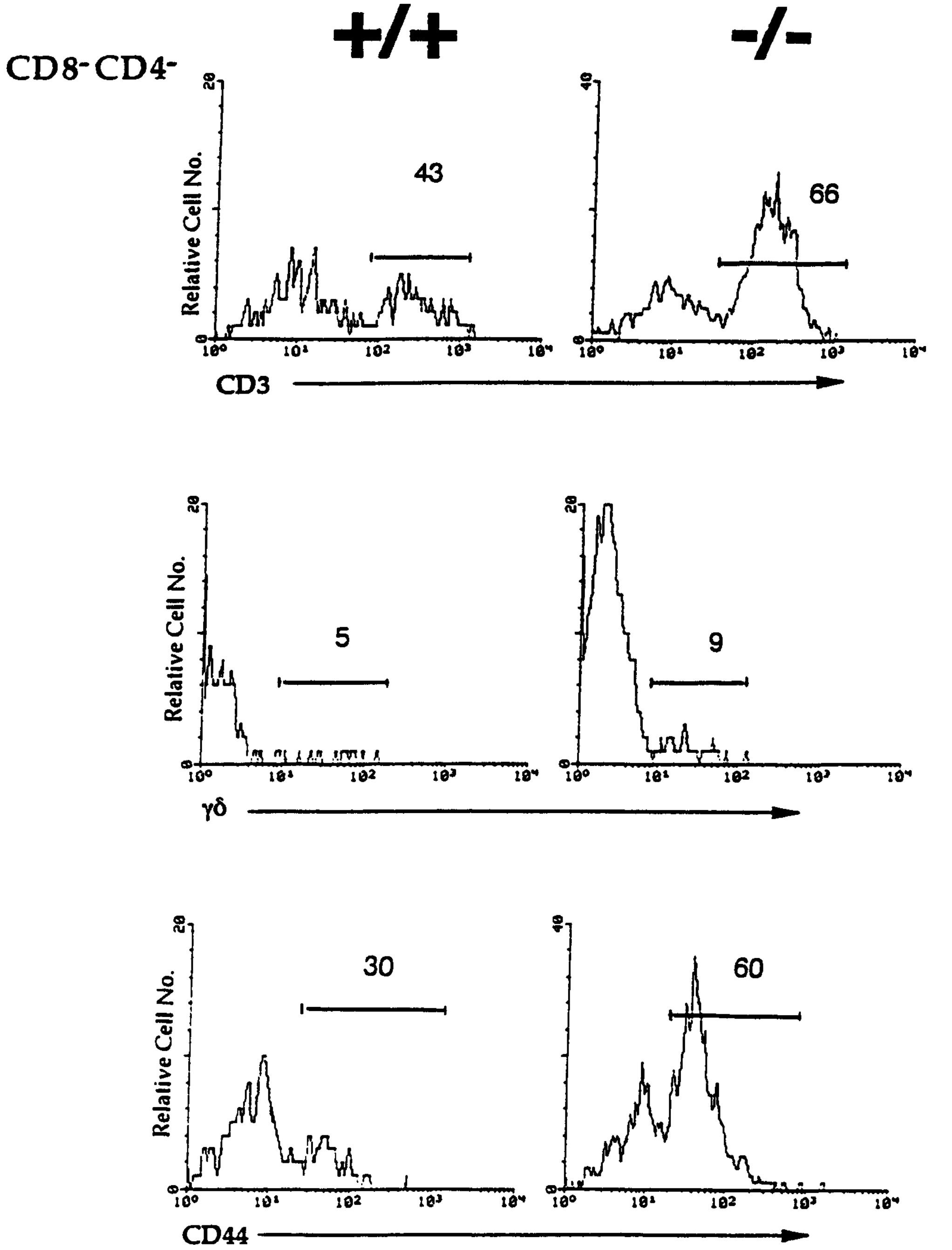

FIGURE 2 Analysis of CD4$^{-}$8$^{-}$ (double negative; DN) thymocytes in IL-2$^{-/-}$ mice. Thymocytes from 5-week-old SPF IL-2$^{-/-}$ (−/−) and IL-2$^{+/+}$ (+/+) mice were stained with PE-conjugated anti-CD4, FITC-conjugated anti-CD8 and either biotinylated anti-CD3, anti-TCR $\gamma\delta$, or anti-CD44 and strepavidin Red 670. DN thymocytes were reanalyzed for expression of CD3 (upper histogram), $\gamma\delta$ cells (middle histogram), and CD44 (lower histogram). The values shown represent the frequency of positive cells. The results shown are representative of those obtained from eight IL-2$^{-/-}$ and seven IL-2$^{+/+}$ mice.

grossly and histologically normal (data not shown). Immunoflourescence and flow cytometric analysis of thymocyte and spleenz-cell populations from reconstituted animals revealed that donor-derived cells comprized approximately 3% of thymocytes and 10-15% of splenic T cells (data not shown) present in the host. There was no significant difference in the degree of chimerism using thymocytes from IL-$2^{-/-}$ versus IL-$2^{+/+}$ mice.

Importantly, thymocytes from the IL-$2^{-/-}$ mice exhibited normal developmental potential as determined by the generation of DP and SP thymocytes (Fig. 3), which was comparable to that seen from IL-$2^{+/+}$ thymocytes. TCR$\alpha\beta^{+}$ and TCR$\gamma\delta^{+}$ thymocytes were present in normal proportions in the thymuses of animals receiving IL-$2^{+/+}$ or IL-$2^{-/-}$ thymocytes. These results demonstrate that the aberrant T-cell development that occurs in IL-$2^{-/-}$ mice does not occur in a normal, intact thymic microenvironment, suggesting that the defects may be caused by microenvironmental defects in the IL-$2^{-/-}$ mice. Consistent with this interpretation was the "normal" phenotype of IL-$2^{-/-}$ splenic T cells generated in a wild-type thymus that, unlike the splenic and lymph node T cells that develop in IL-$2^{-/-}$ thymuses and mice, did not express the activation-associated antigen CD44 (data not shown).

### The Absence of IL-2 Causes a Disruption in Thymic Architecture and Thymic Stromal-Cell Abnormalities

Since thymocyte development is dependent on the interaction with thymic stromal cells, we examined

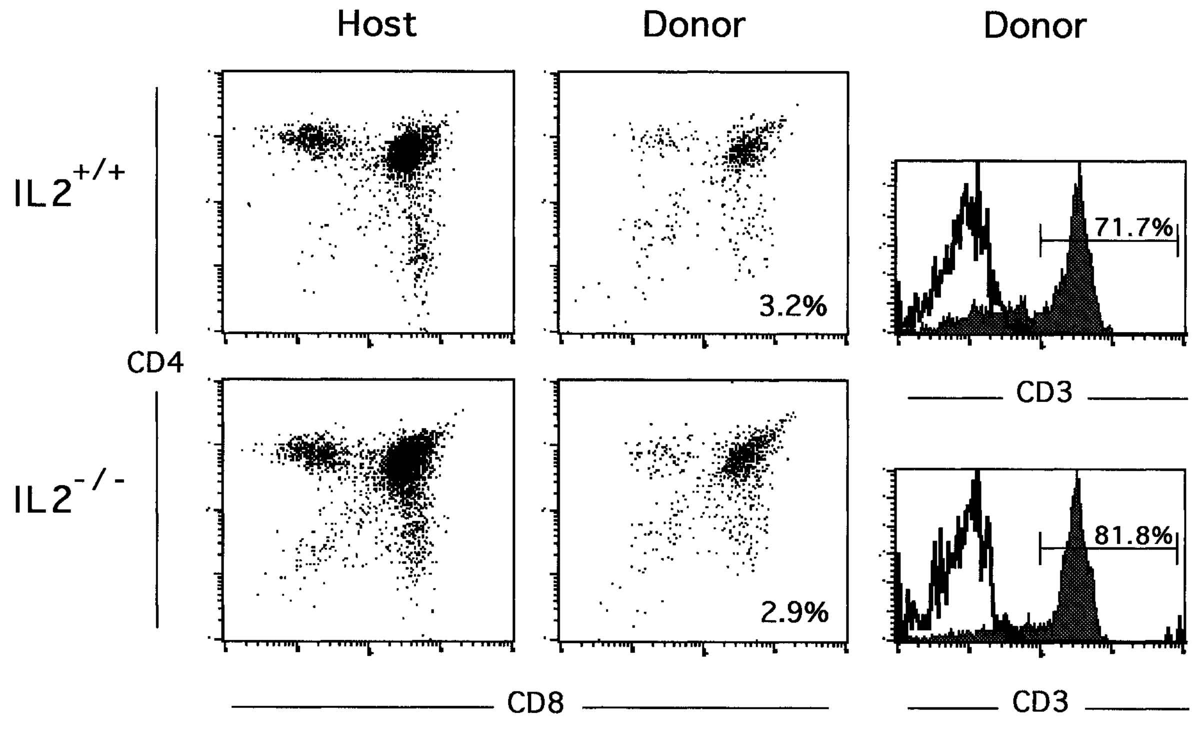

FIGURE 3 $CD3^{-}4^{-}8^{-}$ (triple negative; TN) thymocytes from IL-$2^{-/-}$ mice can develop normally on transfer to the thymus of wild-type mice. Two hundred thousand Thy $1.2^{+}$ TN from 4-week-old SPF IL-$2^{-/-}$ (−/−) or IL-$2^{+/+}$ (+/+) littermates (donor cells) were injected into the thymuses of Thy $1.1^{+}$ C57BL.6 mice (host), and 4 to 5 weeks later, the animals were euthanized and the thymuses analyzed for the presence of CD4-, CD8-, and CD3-expressing cells that were Thy $1.2^{+}$ (donor) or Thy $1.1^{+}$ (host) using three-color flow cytometric analysis as described in the legend of Fig. 1. The values represent the frequency of positive cells. The results shown are representative of those obtained from four animals.

the possibility that the nonlymphoid elements of the thymic microenvironment were affected by the absence of IL-2, resulting in disrupted lymphopoiesis. An immunohistochemical approach was used to obtain evidence for changes in the number, distribution, morphology, and phenotype of the three different groups of stromal cells in IL-2$^{-/-}$ thymuses (mesenchyme-derived fibroblasts, epithelial cells [EC], and bone-marrow-derived monocytes/macrophages and dendritic cells [DC]). Previously characterized antibodies representative of those specific for each stromal-cell type were used for this study. Thymuses from neonatal (1 to 4 weeks of age) and adult (5 to 8 weeks of age) IL-2$^{-/-}$ and IL-2$^{+/+}$ mice (using at least four thymuses per age group) housed under conventional conditions and 8- to 12-week-old germ-free IL-2$^{-/-}$ and IL-2$^{+/+}$ mice were used for this study.

### Nonhematopoietic Stromal Cell Elements

As noted before, after 4 weeks of age, the thymuses of IL-2$^{-/-}$ mice were smaller and contained fewer thymocytes, particularly cortical thymocytes, than those from IL-2$^{+/+}$ littermates. Staining with the medullary and cortical EC-specific antibodies, ER-TR5 and ER-TR4, respectively (van Vliet et al., 1984), revealed abnormal architecture in the IL-2$^{-/-}$ thymus (Fig. 4). In contrast to thymuses from IL-2$^{+/+}$ mice, the medulla was predominant in the thymuses of IL-2$^{-/-}$ mice (Fig. 3). In sections of IL-2$^{-/-}$ thymuses, the ER-TR4$^{+}$ cells were intensely stained and restricted to the outer edges of the thymus, comprising a thin band of positive cells extending from the subcapsule into the medulla. A similar change in the appearance of cortical EC was also seen in the thymuses of 12-week-old germ-free IL-2$^{-/-}$ mice (data not shown). Of note, the loss of ER-TR4$^{+}$ cortical EC in the IL-2$^{-/-}$ thymuses paralleled the loss of immature DN and DP thymocytes. Microscopic analysis of stained cells at higher magnification revealed that ER-TR4$^{+}$ cells in IL-2$^{-/-}$ thymuses were also morphologically different from those in the IL-2$^{+/+}$ thymuses (Fig. 4), being more voluminous and intensely stained. Since the extent of cellular heterogeneity of ER-TR4$^{+}$ cells or the biochemical nature of the antigen(s) recognized by this antibody is not known, the significance of this change in cortical EC morphology and phenotype remains unclear. These changes in the appearance and distribution of thymic cortical EC were first evident between 4 and 5 weeks of age.

The medullary, ER-TR5$^{+}$, EC in the IL-2$^{-/-}$ thymi were indistinguishable from those in IL-2$^{+/+}$ thymuses, which appeared as dense interwoven networks of large semireticular cells with short cytoplasmic processes (Fig. 4). Although the number and morphology of medullary EC was not affected by the absence of IL-2, there was a progressive loss of MHC class II expressing cells in the medulla. This loss resulted in the virtual absence of any MHC class II-expressing cells by 6 to 8 weeks of age (Fig. 4). Since medullary EC represent a major MHC class II$^{+}$ population in the medulla, this observation suggests that IL-2 can regulate the expression of MHC calss II on medullary. By contrast, MHC class I expression was unaffected in newborn, neonatal, and adult IL-2$^{-/-}$ mice, with the distribution and level of expression being equivalent to that seen in the thymuses of IL-2$^{+/+}$ littermates (data not shown). The loss of MHC class II molecules on thymic stromal cells was first evident in mice at approximately 5 weeks of age EC. (data not shown).

Staining with the mesenchymal cell-specific antibody ER-TR7 revealed no obvious differences in the distribution of fibroblasts in the thymuses of 1- to 4-week-old IL-2$^{-/-}$ and IL-2$^{+/+}$ mice (data not shown). The thymuses of 5- to 8-week-old IL-2$^{-/-}$ mice did, however, contain more TR7$^{+}$ cells, and staining with the Picro-Ponceau cytochemical stain showed increased deposition of collagen within the subcapsular layer and underlying cortex consistent with increased fibroblast proliferation (data not shown).

### Stromal Cells of Hematopoietic Cell Origin

Another striking feature of the thymic stroma of conventional (5- to 8-week-old) and germ-free (8- to 12-week-old) IL-2$^{-/-}$ mice was the loss or absence of

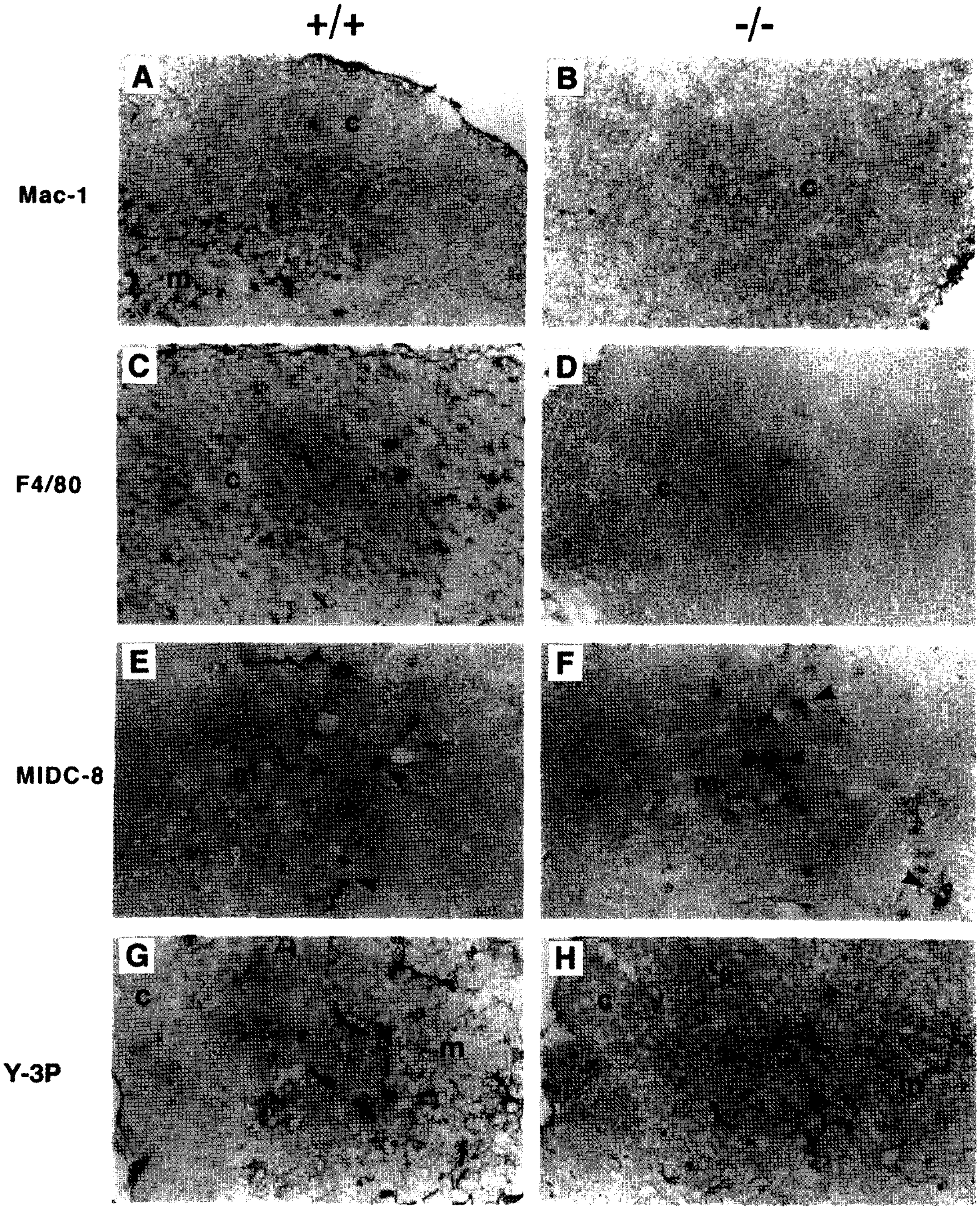

FIGURE 5 Loss of MHC class II expression and bone-marrow-derived stromal cell elements in the thymus of IL-2$^{-/-}$ mice. Frozen sections of thymuses from 5-week-old SPF IL-2$^{+/+}$ (+/+) and IL-2$^{-/-}$ (−/−) mice were stained with the anti-macrophage-specific antibodies Mac-1 (A and B) and F4/80 (C and D), and with the anti-thymic dendritic-cell antibody MIDC-8 (E and F) and the anti-MHC class II-specific antibody Y-3P (G and H). Bound antibody was visualized as described in the legend to Fig. 4. The arrow heads in E and F identify positively stained dendritic cells. m: medulla; c: cortex. Magnification: 200×.

bodies used was due to the downmodulation or loss of surface antigens. Analysis of the other major bone-marrow-derived component of the thymic stroma, dendritic cells (DC), detected no discernible differences in the number or distribution of cells staining with the DC-specific antibody, MIDC-8 (Breel et al., 1987), in the thymuses of SPF (Fig. 4) and germ-free (data not shown) IL-$2^{-/-}$ and IL-$2^{+/+}$ mice. However, since thymic DC normally show strong expression of MHC class II molecules and in view of the reduced level of MHC class II staining in IL-$2^{-/-}$ thymuses the functional properties of thymic DC may be adversely affected by the absence of IL-2.

Changes in the number and distribution of thymic macrophages were first evident in the thymuses of 6-day-old IL-$2^{-/-}$ mice, which already contained fewer cortical and medullary F4/80$^+$ cells (Fig. 6). As the IL-$2^{-/-}$ mice aged, the loss or absence of macrophages became more severe, particularly in the medulla, which by 3 to 4 weeks was essentially devoid of any Mac-1-, Mac-3-, and/or F4/80-expressing cells.

### Thymic Stromal Cells in Germ-Free IL-$2^{-/-}$ Mice

Since germ-free IL-$2^{-/-}$ mice develop disorders of the hematopoietic and immune systems similar to that seen in specific pathogen-free IL-$2^{-/-}$ mice (Contractor et al., in press), we evaluated them for the thymic stromal-cell abnormalities seen in SPF IL-$2^{-/-}$ mice. Changes in cellularity and composition of the thymuses of germ-free IL-$2^{-/-}$ mice were detected after 8 weeks of age (Table I), coincident

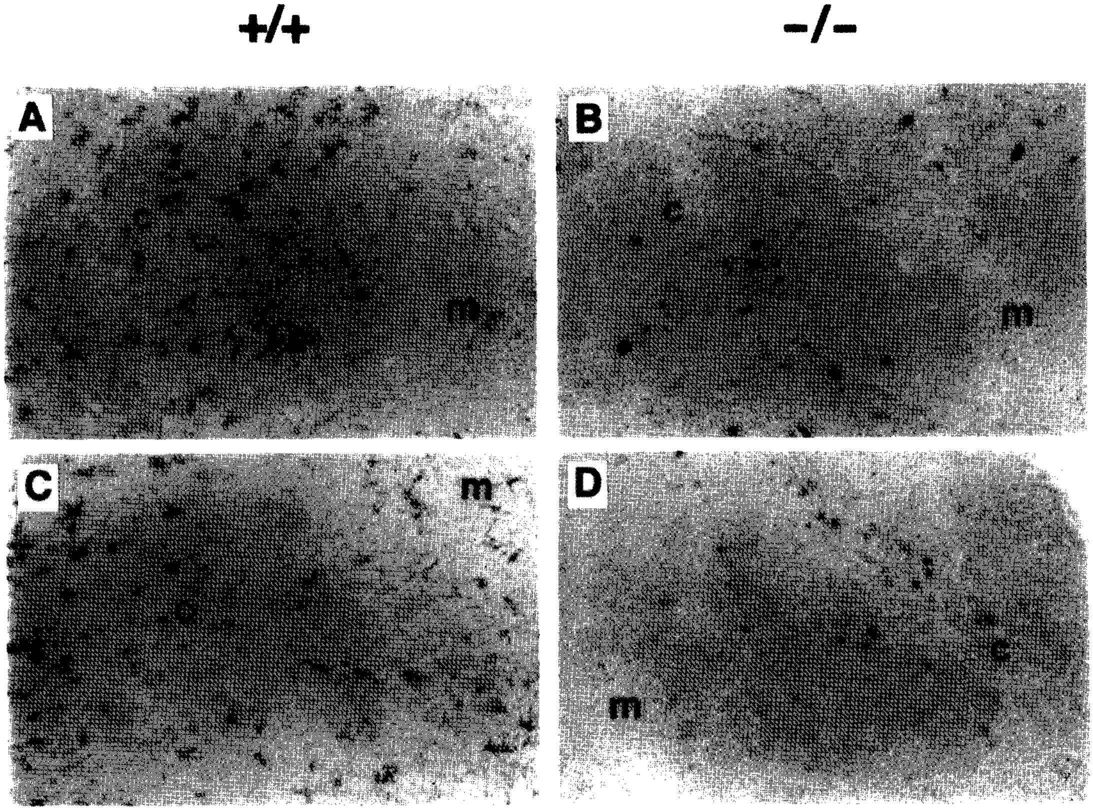

FIGURE 6 Loss of macrophages in the thymuses of neonatal IL-$2^{-/-}$ mice. Frozen sections of thymuses from 6-day-old SPF IL-$2^{+/+}$ (+/+) and IL-$2^{-/-}$ (−/−) mice were stained with the F4/80 antibody as described in the legend to Fig. 5. The results shown represent F4/80 staining of thymuses from two IL-$2^{+/+}$ (A and C) and two IL-$2^{-/-}$ (B and D) littermates. m: medulla; c: cortex. Magnification: 200×.

with the appearance of other signs of disease (e.g., anemia). Changes in thymic stromal-cell populations were evident at 6 weeks of age (Fig. 7), and as seen in the thymuses of SPF IL-2$^{-/-}$ mice, bone-marrow-derived macrophages were particularly affected. Cortical epithelial cells were also reduced in number, and the level of MHC class II staining was also reduced compared to that seen in sections of thymuses from germ-free IL-2$^{+/+}$ animals (data not shown).

In summary, the absence of IL-2 results in the disruption of T-cell development, which appears to be attributable to changes in thymic architecture and deficiencies in specific stromal-cell populations.

### DISCUSSION

The fatal immunopathology that develops in IL-2$^{-/-}$ mice is dependent on the thymic generation of T cells carrying the IL-2 mutation (Krämer et al., 1995), consistent with many previous studies demonstrating a role for IL-2 in normal thymocyte development (reviewed in Carding and Reya, 1994). The way in which IL-2 influences T-cell development and the underlying basis of dysfunctional thymocyte development in IL-2$^{-/-}$ mice is, however, unknown. The results of our analyses of thymic development in IL-2$^{-/-}$ mice have identified abnormalities and deficiencies within populations of stromal cells that we interpret as being the primary cause of dysregulated T-cell development in IL-2$^{-/-}$ mice.

The rescue of IL-2$^{-/-}$ T-cell-progenitor cell development on transfer to wild-type thymuse suggests that these cells are not intrinsically defective and that they can undergo normal IL-2-independent development. Instead, the occurrence of stromal-cell defects and deficiencies in the thymi of IL-2$^{-/-}$ mice

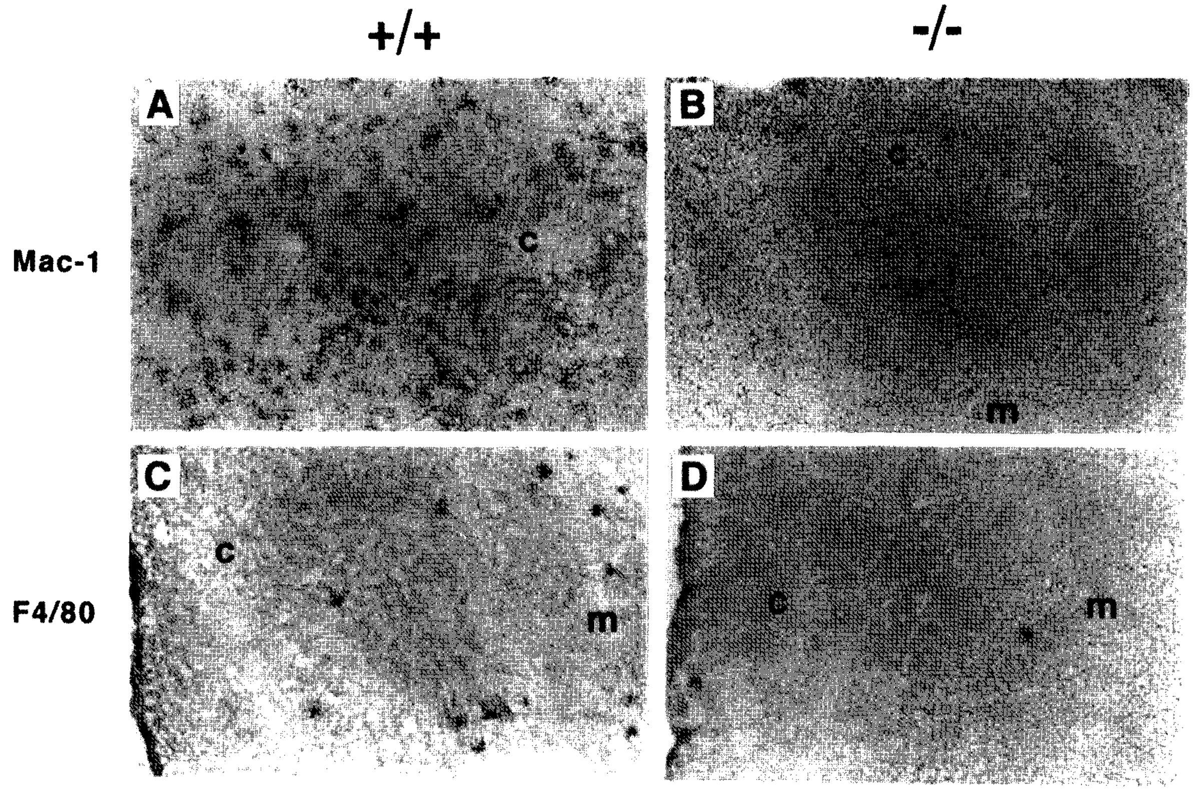

FIGURE 7 Loss of macrophages in the thymus of germ-free IL-2$^{-/-}$ mice. Frozen sections of thymuses from 6-week-old germ-free IL-2$^{+/+}$ (+/+) and IL-2$^{-/-}$ (−/−) mice were stained with the anti-macrophage-specific antibodies Mac-1 (A and B) and F4/80 (C and D), and bound antibody was visualized as described in the legend to Fig. 4. m: medulla; c: cortex. Magnification: 200×.

are consistent with abnormal microenvironments being responsible for dysfunctional thymocyte development in these animals. Although few reagents are available to analyze thymic stromal cells, our studies suggest that the disruption of thymic microenvironments is attributable to the presence of abnormal populations of cortical stromal cells, a progressive loss of MHC class II expression, and bone-marrow-derived macrophages. Together, these defects would severely compromise T-cell repertoire selection and could account for the presence of autoreactive $CD4^+$ T cells and autoimmunity in IL-$2^{-/-}$ mice. The presence of thymic stromal-cell abnormalities in germ-free mice, in which the appearance of hyperactivated T cells is delayed, suggests that the stromal-cell abnormalities are unlikely to be a consequence of the activity of dysfunctional T cells. The failure to detect any effect of the absence of IL-2 on thymic repertoire selection of a MHC class I-restricted CD8 T cells (Krämer et al., 1994) is not surprising in view of the normal distribution of MHC class I expression in the thymuses of IL-$2^{-/-}$ mice (Fig. 3). Yet, the possibility that IL-2/IL-2R signaling is involved in CD4 T-cell repertoire selection cannot be excluded. Our recent finding that thymocytes from ovalbumin-specific, I-$A^d$-restricted, TCR-transgenic mice treated with antigen produce IL-2 while undergoing negative selection and the deletion of antigen-specific $CD4^+CD8^+$ thymocytes *in vivo* is abrogated in the presence of anti-IL-2R antibodies (Bassiri et al., submitted for publication) are consistent with IL-2/IL-2R interactions being directly involved in clonal deletion of MHC class II-restricted thymocytes.

The changes in thymic stromal cells in IL-$2^{-/-}$ mice could, in principle, be attributable to either an intrinsic defect and direct requirement for IL-2, or extrinsic factors and indirect effects of the absence of IL-2. A direct requirement for IL-2 by bone-marrow-derived thymic stromal cells is suggested by the finding that IL-2 is required for growth and development of myeloid cells and its absence in IL-$2^{-/-}$ mice results in abnormal development of granulocytes and tissue-specific macrophages (Reya et al., 1998). In addition, the finding that functional IL-2Rs are normally expressed by Mac-$1^+$ (Thy $1^-$, $CD4^-$, $CD8^-$) MHC class $II^+$ phagocytic (macrophage) cells (Rocha et al., 1988) and that IL-2 can promote their growth and interaction with DN and cortical thymocytes (El Rouby et al., 1985; El Rouby and Papiernik, 1987) are consistent with this hypothesis. Requirement of cytokines produced by thymic macrophages for normal thymocyte development could also explain the breakdown in IL-$2^{-/-}$ thymocyte progenitor development.

It has been proposed that the hematopoietic and immune system disorders that develop in IL-$2^{-/-}$ mice are caused by lymphoid hyperplasia and T-cell infiltration into various tissues (Krämer et al., 1995; Sadlack et al., 1995). Although we cannot exclude the possibility that dysfunctional T cells contribute to or exacerbate the thymic defects in these mice, the fact that stromal-cell defects are apparent in neonatal IL-$2^{-/-}$ mice that have very few peripheral T cells and show no signs of disease makes it unlikely they are the cause of this disorder.

Since the cortical and medullary macrophage populations are initially intact in the thymuses of neonatal (1- to 3-day-old) IL-$2^{-/-}$ mice, IL-2 may function primarily as a survival factor for these cells. Consistent with such an effect of IL-2 on myeloid cells is its ability to promote the survival *in vitro* of mature neutrophils (Pericle et al., 1994). A differential requirement for IL-2 by thymic macrophages during neonatal and adult stages of T-cell development is another attractive possibility in light of other studies demonstrating age-related changes in both the developmental potential of macrophage progenitor populations and the microenvironments in which they develop (Naito, 1993; Naito et al., 1996). The observation that other bone-marrow-derived stromal cells (dendritic cells) are intact in IL-$2^{-/-}$ thymuses suggests that the loss of thymic macrophages is not related to stress and the systemic production of corticosteroids. Although the expression of functional IL-2Rs by EC has been described (Weidmann et al., 1992; Ciacci et al., 1993), we have been unable to detect expression of IL-2R-mRNA or cell-surface protein in freshly isolated or primary cultures of thymic EC (SRC unpublished observations). The changes seen among cortical epithelial cells in the IL-

2$^{-/-}$ thymuses may, therefore, be a consequence of indirect effects of the absence of IL-2. The ability of thymocytes to influence the growth of thymic epithelial cells by direct cell contact (Surh et al., 1992; Palmer et al., 1993) and through the production of growth factors (Carding and Reya, 1994) may explain the presence of abnormal epithelial cells in the thymuses of IL-2$^{-/-}$ mice. Regardless of the causes of these changes, the progressive loss of macrophages and disruption of thymic stromal microenvironments provide an explanation why T-cell development is initially intact in neonatal IL-2$^{-/-}$ mice and autoimmunity is only apparent in older animals.

Although our findings are most consistent with abnormalities in thymic stromal cells being responsible for the disruption in thymocyte development in IL-2$^{-/-}$ mice, we cannot entirely exclude the possibility that IL-2 may also be required for thymocyte growth and development. Although the rescue of IL-2$^{-/-}$ T-cell-progenitor cell development on transfer to wild-type thymuses suggests that IL-2 is not required for T-cell-progenitor development, it is possible that their development is influenced by the IL-2 produced by the wild-type, host-derived, DN thymocytes (Yang-Snyder and Rothenberg, 1993; Zlotnik and Kelner, 1994) that are also present in the reconstituted thymuses. Since cortical thymocytes are susceptible to corticosteroid-induced death, it is possible that stress and steroid production may contribute to, or exacerbate, the disruption in thymocyte development and loss of DP thymocytes. Additional experiments are required to address these possibilities.

In summary, our data demonstrate that IL2 is required in order to promote the development and maintenance of the nonlymphoid stromal-cell populations that comprise the thymic microenvironments necessary for the support of T-cell-progenitor cell growth and development.

### MATERIALS AND METHODS

### Mice

C57BL.6-Thy.1 (B6.PL-*Thy1$^a$*/Cy)-congenic mice were obtained from the Jackson Laboratory (Bar Harbor, ME) and were used between 4 and 6 weeks of age. Specific pathogen-free (SPF) IL-2$^{-/-}$ mice backcrossed for eight generations to C57BL.6 were obtained from R. Schwartz (NIH, Bethesda, MD). The mice were bred and maintained in individual filter cages at the University of Pennsylvania and were used between 1 and 7 weeks of age. Sentinel animals housed with the mutant mice were routinely screened for the presence of common mouse pathogens and none has been detected at any time since the animals have been in our facility. Eight- to twelve-week-old gnotobiotic (germ-free) IL-2$^{-/-}$ and IL-2$^{+/+}$ and IL-2$^{+/-}$ mice were rederived from our SPF colony of IL-2$^{-/-}$ mice by Taconic Farms (Germantown, NY) and were certified as being free of any viral and/or bacterial organisms. The mice were fed a sterile (autoclaved) mouse diet (PMI Feeds, St Louis) and were housed in isolator cages within a flexible film isolator in the gnotobiotic facility of the Biology Department at the University of Pennsylvania. The germ-free status of all animals used was verified by bacteriological, histological, and serological analyses of tissues and fluids at autopsy. Homozygous animals were derived from homozygote (germ-free animals) or heterozygote (SPF and germ-free animals) matings and were identified from samples of tail DNA by PCR (Schorle, 1991). Heterozygote sex-matched littermates were used as controls for all experiments using IL-2$^{-/-}$ animals or cells. The frequency and age of onset of anemia and colitis in homozygous animals maintained in our facility were the same as previously reported (Sadlack et al., 1993).

### Cell Isolation and Intrathymic Transfer

CD4$^-$, CD8$^-$, CD3$^-$ (triple negative; TN) thymocytes from IL-2$^{-/-}$ and IL-2$^{+/+}$ (Thy 1.2$^+$) mice were isolated using a negative immunomagnetic isolation procedure (Belles et al., 1996) incorporating anti-CD4 (clone GK1.5; American Tissue Type Culture Collection [ATCC], Rockville, MD), anti-CD8 (TIB210; ATTC), and anti-CD3 (29B; Life Technologies, Gaithersburg, MD), and goat anti-mouse/rat Ig-coated immunomagnetic particles (BioMag Beads, Perseptive Diagnostics, Cambridge, MA). All incubations were carried on ice for 15 to 20 min to minimize any downmodulation of surface antigens. The purity of the selected cell population was verified by flow cytometry and was routinely > 98% $CD4^-8^-$ and > 92% $CD3^-$ (Fig. 1) with the residual CD3-expressing cells being $CD3^{lo}$, similar to immature $CD3^+$ DN thymocytes (Fig. 1). Between 1 and 2 × $10^5$ IL-$2^{-/-}$ or IL-$2^{+/+}$ TN thymocytes were injected in a volume of 10 to 20 $\mu$l of PBS into each lobe of the thymuses of sublethally irradiated (650 R) Thy $1.1^+$ C57BL.6 mice. Approximately, 4 weeks later, animals were euthanized and the thymus and spleen analyzed for donor cells by flow cytometry using antibodies reactive with the donor-specific allele of Thy 1 (Thy 1.2).

### Flow Cytometry

The following mouse and rat monoclonal antibodies were used to stain and analyze thymocytes isolated from IL-$2^{-/-}$ and IL-$2^{+/+}$ mice. CD90/Thy 1.2 (30H.12; Life Technologies); CD90/Thy1.1 (HIS51; Pharmingen, San Diego); CD3 (29B; Life Technologies); CD4 (CT-CD4; Caltag Laboratories, San Francisco); CD8$\alpha$ (5.3-6.7; Life Technologies); CD24/HSA (M1/69; Pharmingen); CD25/IL-2R$\alpha$ (PC61; ATCC); CD44/Pgp-1 (IM7; Pharmingen); CD69 (H1.2F3; Pharmingen); CD122/IL-2R$\beta$ (TM$\beta$1; Pharmingen); TCR$\alpha\beta$ (H57-597; Pharmingen), TCR$\gamma\delta$ (GL3; Pharmingen). Stepavidin-phycoerythrin and strepavidin-Red670 (SA-PE, SA-Red670; Life Technologies) were also used to detect reactivity of biotinylated primary antibodies. Antibody staining and flow cytometric analysis were carried out using a FACScan and Lysis II or CellQuest software (Becton-Dickinson, San José, CA), as described previously (Reya et al., 1996).

### Immunohistochemistry

Frozen sections (5 $\mu$m) of newborn (1- to 3-day-old), neonatal (1- to 3-week-old), and adult (4- to 7-week-old) IL-$2^{-/-}$ and IL-$2^{+/+}$ thymuses were fixed in ice-cold acetone, incubated with blocking solution (5 $\mu$g/ml mouse IgG [Sigma, St. Louis]/5% normal rat serum/5% normal rabbit serum in 10 mM Tris, pH 7.3, 0.15 M NaCl, and 0.02% Triton ×100 [TBST]) prior to incubation with primary antibodies. At least four independently obtained thymus samples from IL-$2^{-/-}$ and IL-$2^{+/+}$ mice of each age group were used for this analysis. Optimal concentrations of monoclonal antibodies reactive with cortical (ER-TR4; van Vliet et al., 1984) or medullary (ER-TR5; van Vliet et al., 1984) epithelial cells, fibroblasts, and other connective tissue elements (ER-TR7; van Vliet et al., 1984), monocytes/macrophages (CD11b/Mac-1 [M1/70], Caltag; F4/80, Caltag; Mac-3, Pharmingen), interdigitating dendritic cells (MIDC-8; Serotec, Oxford, UK), or MHC class I (Y-3; ATCC) and II (Y-3P; ATCC) molecules were prepared in TBST and incubated with sections at 20°C for 60 min. Isotype-matched rat and mouse IgG antibodies of irrelevant specificity were used as controls. The sections were washed with TBST and incubated with either a biotin-labeled mouse anti-rat IgG (Vector Laboratories, Burlingame, CA) or biotin-labeled rabbit anti-mouse IgG (Vector) monoclonal antibody, washed, and incubated with a biotin-avidin-alkaline phosphatase complex (Vector). The Vector Red (Vector) alkaline phosphatase substrate kit was used to visualize antibody-labeled cells and sections were counterstained with methyl green prior to mounting and photomicroscopy.

### *Acknowledgments*

Tannishta Reya and Hamid Bassiri contributed equally to this work. This work was supported by grants from the American Cancer Society (RPG-97-027), the NIH (AI31972), and the University of Pennsylvania Research Foundation.

### *References*

Bassiri H., Egan P.E., Samoilova E., Chen Y. and Carding S.R. (Submitted for publication). Interleukin 2 is required for the induction of apoptosis in developing T cells and the deletion of MHC class II-restricted thymocytes.

Belles C., Kuhl A.L., Donoghue A., Sano Y., O'Brien R.L., Born W., Bottomly K. and Carding S.R. (1996). Bias in the $\gamma\delta$ T cell response to *Listeria monocytogenes*. J. Immunol. **156**:4280-4289.

Breel M., Mebius R.E. and Kraal G. (1987). Dendritic cells of the mouse recognized by two monoclonal antibodies. Eur. J. Immunol. **17**:1555-1559.

Carding S.R. and Reya T. (1994). Cytokines in the fetal thymus. In Intrathymic T-cell development, Nikolic-Zugic, J., ed. (Austin, TX: R. G. Landes), pp. 46-63.

Ciacci C., Mahida R., Dignass A., Koizumi M. and Podolsky D.K. (1993). Functional interleukin-2 receptors on intestinal epithelial cells. J. Clin. Invest. **92**:527-532.

Contractor N.V., Bassiri H., Reya T., Park A.V., Baumgart D.C., Wasik M., Emerson S.G. and Carding S.R. (In press). Lymphoid hyperplasia, autoimmunity and compromised intestinal intraepithelial lymphocyte development in colitis-free gnotobiotic interleukin 2-deficient mice. J. Immunol.

El Rouby S. and Papiernik M. (1987). ConA response and interleukin-2 receptor expression of thymocytes rosetting with phagocytic cells of the thymus. Thymus **9**:239-246.

El Rouby S., Praz F., Halbwachs-Mecarelli L. and Papiernik M. (1985). Thymic reticulum in mice. IV. The rosette formation between phagocytic cells of the thymic retisulum and cortical typr thymocytes is mediated by complement receptor type 3. J. Immunol. **134**:3625-3632.

Godfrey D.I. and Zlotnik A. (1993). Control points in early T cell development. Immunol. Today **14**:547-553.

Hanke T., Mitnacht R., Boyd R. and Hünig T. (1994). Induction of interleukin-2 receptor $\beta$-chain expression by self-recognition in the thymus. J. Exp. Med. **180**:1629-1636.

Horak I., Löhler J., Ma A. and Smith K.A. (1995). Interleukin-2 deficient mice: A new model to study autoimmunity and self-tolerance. Immunol. Rev. **148**:35-43.

Hume D.A., Robinson A.P. and Macpherson G.G. (1983). The mononuclear phagocyte system of the mouse defined by immunohistochemical localization of antigen F4/80: Relationship between macrophages, Langerhans cells, reticular cell and dendritic cells in lymphoid and hematopoietic organs. J. Exp. Med. **158**:1522-1536.

Jenkinson E.J., Kingston R. and Owen J.J.T. (1987). Importance of IL-2 receptors in intra-thymic generation of cells expressing T-cell receptors. Nature **329**:160-162.

Krämer S., Mamalaki C., Horak I., Schimpl A., Kioussis D. and Hünig T. (1994). Thymic selection and peptide-induced activation of T cell receptor-transgenic CD8 T cells in interleukin-2-deficient mice. Eur. J. Immunol. **24**:2317-2322.

Krämer S., Schimpl A. and Hunig T. (1995). Immunopathology of interleukin (IL) 2-deficient mice: Thymus dependence and supression by thymus-dependent cells with an intact IL-2 gene. J. Exp. Med. **182**:1769-1776.

Lúdvíksson B.R., Gray B., Strober W. and Ehrhàrdt R.O. (1997). Dysregulated intrathymic development in the IL2-deficient mouse leads to colitis-inducing thymocytes. J. Immunol. **158**:104-111.

Naito M. (1993). Macrophage heterogeniety in development and differentiation. Arch. Histol. Cytol. **56**:331-351.

Naito M., Umeda S., Yamamoto T., Moriyama H., Umezu H., Hasegawa G., Usuda H., Shultz L.D. and Takahashi K. (1996). Development, differentiation and phenotypic heterogeniety of murine tissue macrophages. J. Leuk. Biol. **59**:133-138.

Palmer D.B., Viney J.L., Ritter M.A., Hayday A.C. and Owen M.J. (1993). Expression of the $\alpha\beta$ T-cell receptor is necessary for the generation of the thymic medulla. Dev. Immunol. **3**:175-179.

Pericle F., Liu J.H., Diaz J.I., Blanchard D.K., Wei S., Forni G. and Djeu J.Y. (1994). Interleukin-2 prevention of apoptosis in human neutrophils. Eur. J. Immunol. **24**:440-444.

Reya T., Contractor N.V., Couzens M.S., Wasik M.A., Emerson S.G. and Carding S.R. (in press). Abnormal myelocytic cell development in interleukin-2 (IL2)-deficient mice: Evidence for the involvement of IL2 in myelopoiesis blood.

Reya T., Yang-Snyder J.A., Rothenberg E. V. and Carding S.R. (1996). Regulated expression and function of CD122 (interleukin-2/intereukin-15R-$\beta$) during lymphoid development. Blood **87**:190-201.

Rocha B., Lehuen A. and Papiernik M. (1988). IL2-dependent proliferation of thymic accessory cells. J. Immunol. **140**:1076-1080.

Sadlack B., Löhler J., Schorle H., Klebb G., Haber H., Sickel E., Noelle R.J. and Horak I. (1995). Generalized autoimmune disease in interleukin-2-deficient mice is triggered by an uncontrolled activation and proliferation of CD4$^{+}$ T cells. Eur. J. Immunol. **25**:3053-3059.

Sadlack B., Merz H., Schorle H., Schimpl A., Feller A.C. and Horak I. (1993). Ulcerative colitis-like disease in mice with a disrupted interleukin-2 gene. Cell **75**:253-261.

Schorle H., Holtschke T., Hunig T., Schimpl A. and Horak I. (1991). Development and function of T cells in mice rendered interleukin-2 deficient by gene targeting. Nature (London) **352**:621-624.

Skinner M., Le Gros G., Marbrook J. and Watson J.D. (1987). Development of fetal thymocytes in organ culture: Effect of IL2. J. Exp. Med. **165**:1481-1493.

Surh C.D., Ernst B. and Sprent J. (1992). Growth of epithelial cells in the thymic medulla is under the control of mature T cells. J. Exp. Med. **176**:611-616.

Tanaka T., Takeuchi Y., Shiohara T., Kitamura F., Nagasaka Y., Hamamura K., Yagita H. and Miyasaka M. (1992). *In utero* treatment with monoclonal antibody to IL-2 receptor $\beta$-chain completely abrogates development of Thy-1$^{+}$ dendritic epidermal cells. Int. Immunol. **4**:487-491.

Tentori L., Longo D.L., Zuniga-Pflucker J.C., Wing C. and Kruisbeek A.M. (1988). Essential role of the interleukin 2-interleukin 2 receptor pathway in thymocyte maturation in vivo. J. Exp. Med. **168**:1741-1747.

Van Ewijk W. (1988). Cell surface topography of thymic microenvironments. Lab. Invest. **59**:579-590.

Van Vliet E., Melis M. and van Ewijk W. (1984). Monoclonal antibodies to stromal cell types of the mouse thymus. Eur. J. Immunol. **14**:524-529.

Waanders G.A. and Boyd R.L. (1990). The effects of interleukin 2 on early and late thymocyte differentiation in foetal thymus organ culture. Int. Immunol. **2**:461-468.

Waanders G.A., Godfrey D.I. and Boyd R.L. (1989). Modulation of T-cell differentiation in murine fetal thymus organ cultures. Thymus **13**:73-82.

Weidmann E., Sacchi M., Plaisance S., Seog-Heo D., Yasumura S., Lin W.C., Johnson J.T., Herberman R.B., Azzarone B. and Whiteside T.L. (1992). Receptors for interleukin-2 (IL2R) on human squamous cell carcinoma cell lines and tumor in situ. Cancer Res. **52**:5963-5967.

Wekerle H., Ketelsen U.-P. and Eust M. (1980). Thymic nurse cells. Lymphoepithelial complexes in murine thymus. Non-Lymphoid serological characterization. J. Exp. Med. **151**:925-944.

Yang-Snyder J.A. and Rothenberg E.V. (1993). Developmental and anatomical patterns of IL-2 gene expression *in vivo* in the murine thymus. Dev. Immunol. **3**:85-102.

Zlotnik A. and Kelner G.S. (1994). Cytokines in the adult thymus. In Intrathymic T-Cell Development, Nikolic-Zugic, J., ed. (Austin, TX: R. G. Landes), pp. 64-75.

Zuniga-Pflucker J.C. and Kruisbeek A.M. (1990). Intrathymic radioresistant stem cells follow an IL-2/IL-2R pathway during thymic regeneration after sublethal irradiation. J. Immunol. **144**:3736-3740.

Zuniga-Pflucker J.C., Smith K.A., Tentori L., Pardoll D.M., Longo D.L. and Kruisbeek A.M. (1990). Are the IL-2 receptors expressed in the murine fetal thymus functional? Dev. Immunol. **1**:59-66.

